# CEBPG suppresses ferroptosis through transcriptional control of *SLC7A11* in ovarian cancer

**DOI:** 10.1186/s12967-023-04136-0

**Published:** 2023-05-20

**Authors:** Xiaoqian Zhang, Xiaocui Zheng, Xiang Ying, Weiwei Xie, Yujia Yin, Xipeng Wang

**Affiliations:** Department of Obstetrics and Gynecology, XinHua Hospital, Shanghai JiaoTong University School of Medicine, 1665 Kongjiang Rd, Yangpu District, Shanghai, 200092 China

**Keywords:** CEBPG, Ovarian cancer, Ferroptosis, *SLC7A11*

## Abstract

**Background:**

Ovarian cancer (OC) has high mortality and poor prognosis for lacking of specific biomarkers and typical clinical symptoms in the early stage. CEBPG is an important regulator in tumor development, yet it is unclear exactly how it contributes to the progression of OC.

**Methods:**

TCGA and tissue microarrays with immunohistochemical staining (IHC) were used to examine CEBPG expression in OC. A variety of in vitro assays were conducted, including colony formation, proliferation, migration, and invasion. The orthotopic OC mouse model was established for in vivo studies. Ferroptosis was detected by observing mitochondrial changes with electron microscopy, detecting ROS expression, and detecting cell sensitivity to drugs by CCK8 assay. The interaction between CEBPG and *SLC7A11* was confirmed by CUT&Tag and dual luciferase reporter assays.

**Results:**

A significantly higher expression level of *CEBPG* in OC when compared with benign tissues of ovary, and that high *CEBPG* expression level was also tightly associated with poor prognosis of patients diagnosed with OC, as determined by analysis of datasets and patient samples. Conversely, knockdown of *CEBPG* inhibited OC progression using experiments of OC cell lines and in vivo orthotopic OC-bearing mouse model. Importantly, CEBPG was identified as a new participator mediating ferroptosis evasion in OC cells using RNA-sequencing, which could contribute to OC progression. The CUT&Tag and dua luciferase reporter assays further revealed the inner mechanism that CEBPG regulated OC cell ferroptosis through transcriptional control of *SLC7A11*.

**Conclusions:**

Our findings established CEBPG as a novel transcriptional regulator of OC ferroptosis, with potential value in predicting clinical outcomes and as a therapeutic candidate.

**Supplementary Information:**

The online version contains supplementary material available at 10.1186/s12967-023-04136-0.

## Introduction

Ovarian cancer (OC) is the seventh most common malignant tumor and ranks eighth among the causes of cancer death in female [1]. The latest statistics show that in 2021, the number of deaths from OC in the United States is about 22,950, and its mortality rate ranks first among gynecological malignancies, seriously threatening women’s life and health [2]. As there are no specific biomarkers at the beginning of the disease and no typical clinical symptoms, over 70% of cases have advanced to the late stage when diagnosed. Moreover, following cytoreductive surgery and platinum-based chemotherapy as first-line treatment, some OC patients may develop chemotherapy resistance or relapse [3]. Although many years of research have been carried out, the 5-year survival rate of OC patients is only approximately 45% [4]. As a result, early detection of OC and potential therapeutic targets need to be explored urgently and specifically.

As a member of the leucine zipper transcription factor family, CCAAT/enhancer binding protein gamma, also named CEBPG, has a key role in several biological processes, including energy metabolism, cell differentiation, and proliferation [5, 6]. Recently, CEBPG has gained considerable attention as a potential molecular marker for cancer progression and as a therapeutic target [6, 7]. For instance, *CEBPG* deregulation could result in the differentiation arrest of malignant cells in acute myeloid leukemia [8]. Also, CEBPG could promote the proliferation and migration of by activating PI3K-AKT signaling pathway in esophageal squamous cell carcinoma cells [7]. Moreover, CEBPG has been suggested to be a biomarker for lung cancer risk [9]. However, while multiple studies have revealed CEBPG’s role in promoting tumor growth, there is still a great deal of uncertainty about its role in OC. Therefore, in this study, we intend to further evaluate the specific role of CEBPG in OC progression.

A nonapoptotic form of cell death, ferroptosis is induced by cystine depletion and membrane damage controlled by extensive lipid peroxidation [10]. Dysregulation of ferroptosis is closely linked to tumorigenesis of multiple cancer [11]. And increasing evidence has shown that ferroptosis is a crucial factor in tumor suppression. Therefore, the potential for cancer treatment lies in targeting ferroptosis [12–14]. Solute carrier family 7 member 11 (SLC7A11) is a key cystine transporter, which could transport extracellular cystine into cells for further conversion to glutathione (GSH) [15]. Then, glutathione peroxidase 4 (GPX4) could utilize GSH to reduce lipid hydroperoxides, thereby inhibiting ferroptosis. Existing studies have shown that SLC7A11 is one of the key inhibitors of ferroptosis, and inhibition of SLC7A11 can trigger ferroptosis [15–17]. However, the mechanism by which CEBPG regulates ferroptosis and the relationship between CEBPG and SLC7A11 during OC progression are still little known.

Here, we first used data of OC samples from The Cancer Genome Atlas (TCGA) and OC tissue microarrays to analyze the level of CEBPG in OC compared with benign tissues, and its relationships with clinical outcomes of OC patients. Notably, our results indicated that patients with OC who express high levels of CEBPG have a poorer prognosis. We subsequently established *CEBPG* knockdown cell lines to further explore CEBPG's role in OC cells and in mice. Mechanistically, the results of CUT&Tag and luciferase reporter assays showed that CEBPG inhibited ferroptosis of tumor cells by upregulating SLC7A11, a negative regulator of ferroptosis, thus accelerating the progression of OC. Consequently, it suggested that CEBPG may be used as a biomarker and a potential therapeutic target for OC.

## Materials and methods

### Patients and specimens

Tissues, including 80 ovarian benign cyst tissues, 117 OC tissues, and 69 peritoneal metastatic OC tissues, were collected with informed consent from patients in Xinhua Hospital between 2008 and 2018. The overall survival rate (OS) is measured from the time of surgery until death or last follow-up. Xinhua Hospital's Ethics Committee approved this study of patient specimens.

### Immunohistochemical (IHC) analysis

In a tissue microarray (TMA), 80 ovarian benign cyst tissues, 117 OC tissues, and 69 peritoneal metastatic OC tissues were assembled and analyzed with an anti-CEBPG antibody (12997-1-AP, Proteintech, 1:100 dilution) or an anti-SLC7A11 antibody (A13685, ABclonal, 1:100 dilution).

### Cell culture

We mainly cultured A2780 and Hey, two human OC cell lines obtained from the Chinese Academy of Sciences, in DMEM (Gibco, USA) supplemented with 10% fetal bovine serum (FBS; Gibco, USA) and 1% antibiotics under standard culture conditions (37 °C, 5% CO_2_). Analysis of short tandem repeats (STR) was used to verify the authenticity of all cell lines.

### Plasmid transfection and lentiviral infection

To generate *CEBPG* stable knockdown cell lines, lentiviral vectors with control shRNA or 3 specific shRNAs targeting *CEBPG* were designed by Hanyin Biotechnology Limited Company (China), and the shRNA sequences are listed in Additional file 1: Table S1 of the supplementary materials. After infection (following the instructions of the manufacturer) and testing, shRNA-A and shRNA-C were selected and used to generate *CEBPG* knockdown A2780 *CEBPG*-Sh1/Sh2 and Hey *CEBPG* Sh1/Sh2 cells. The negative control cell lines A2780 *CEBPG*-NC and Hey *CEBPG*-NC were generated by infecting lentiviruses with control shRNA. We overexpressed CEBPG or SLC7A11 by inserting full-length *CEBPG* or *SLC7A11* coding sequences into the pCDH vector and transfecting stable *CEBPG* knockdown cell lines to generate the A2780/Hey *CEBPG*-OE and A2780/Hey *SLC7A11*-OE. Transfection of empty vectors generated negative control cell lines. To re-express *SLC7A11* in stable *CEBPG* knockdown cell lines (A2780/Hey *CEBPG*-sh1 + *SLC7A11*), objective OC cells were infected with lentiviral vectors with full-length *SLC7A11* coding sequences or with the control vector.

### Quantitative real-time PCR (qRT-PCR)

To extract total RNA from cells, TRIzol reagent (Takara, Japan) was used. And then PrimeScript^™^ RT Master Mix (Takara, Japan) was used to reverse transcribe it. The levels of RNA transcripts were quantified using qRT-PCR using the LightCycler 480 Real-Time PCR system and SYBR Green PCR kit (Yeasen Biotechnology, China). qRT-PCR primers are shown in Additional file 1: Table S2 of the supplements. In order to calculate relative gene expression, the comparative threshold cycle method was used.

### Western blotting

RIPA lysis buffer (Solarbio, China) was used to lyse cell pellet firstly. Proteins obtained were then separated using SDS‒PAGE (10% or 12.5% concentration) before transferred to 0.45 μm polyvinylidene fluoride membranes (Millipore, USA). After blocked with BSA (5%) for 2 h at room temperature, the membranes were incubated with primary antibodies at 4 °C overnight. The next day, the membranes were incubated with HRP-conjugated secondary antibodies at room temperature for 1 h before visualized with the chemiluminescent substrate (Millipore, US). Antibodies used were as follows: anti-CEBPG (12997-1-AP, Proteintech, China), anti-SLC7A11 (A13685, ABclonal, China), anti-E-cadherin (20874-1-AP, Proteintech, China), anti-GAPDH (10494-1-AP, Proteintech, China), anti-E-cadherin (20874-1-AP, Proteintech, China), anti-GAPDH (10494-1-AP, Proteintech, China) and anti-Tubulin (11224-1-AP, Proteintech, China).

### CCK8 assay

Proliferation assays were conducted on OC cells in 96-well plates using Cell Counting Kit 8 (CCK8) reagent (Beyotime, China) daily for five days. Incubation took place at 37 °C for 2 h, and the plate was analyzed with a microplate reader at 450 nm in order to measure absorbance.

Viability assays were performed by seeding cells in 96-well plates the following day and treating them with drugs for the appropriate duration of time. Then, fresh medium containing 10% CCK8 reagent was added to replace the drug-containing medium. The absorbance at 450 nm was measured after 2 h of incubation at 37 °C. The negative control was DMSO.

### Colony formation assay

In 6-well plates, 500 cells were seeded per well for 10 days. Colonies were counted after staining with 0.2% crystal violet for 10 min.

### Migration and invasion assays

In a Transwell chamber, cells were suspended in serum-free DMEM and added to the upper compartment with an 8-μm pore size membrane (Corning, USA) (for the migration assay) (BD Biosciences, USA) (for the invasion assay). For the lower compartment, DMEM supplemented with 10% FBS was added. Cells that invaded or migrated into the lower compartment were counted after 24 h with 0.2% crystal violet.

### RNA sequencing

OC cell lines (*CEBPG*-knockdown and negative control A2780 cells) were used for RNA sequencing. We isolated and purified total RNA using TRIzol reagent (Takara, Japan) in accordance with the manufacturer's instructions. After next-generation sequencing (LC-Bio Technologies, China), raw data were analyzed using fastp software. The sequencing data were aligned to the human genome (Homo sapiens, GRCh38) using HISAT2, and.bam files were generated. Genes or transcripts were assembled using StringTie software and quantified with FPKM using the R package edgeR for the analysis of differentially expressed genes between the samples; genes with a fold change of > 2 or < 0.5 and a p value < 0.05 were defined as differentially expressed. Finally, the genes were analyzed using DAVID software for GO enrichment and KEGG enrichment.

### Transmission electron microscopy (TEM)

An ultrastructural analysis of mitochondria was performed using TEM. We treated stable *CEBPG*-knockdown and *CEBPG*-NC A2780 cells with or without erastin, fixed them at 4 °C for 4 h, and then applied 1% OsO4 to them for 2 h at room temperature prior to dehydration and infiltration. The samples were then successively dehydrated, infiltrated, embedded and sliced into ultrathin sections (60–80 nm). TEM (Hitachi, Japan) images of ultrathin sections were taken after staining with uranyl acetate or lead citrate.

### Lipid peroxidation assay

Following seeding cells in 6-well plates, drugs were administered for an appropriate length of time the following day, and incubated with 10 μM DCFH-DA (Yeasen Biotechnology, China) at 37 °C for 30 min. Trypsin digested cells were suspended in PBS after being washed twice with 1 mL of serum-free DMEM. Finally, fluorescence changes were analyzed using a flow cytometer.

### Light microscopy

On the day following cell culture, drugs were added to 6-well plates for an appropriate period of time. Images were captured using a Leica microscope.

### High-throughput CUT&Tag

To explore the molecular function of CEBPG as a transcription factor, cleavage under targets and tagmentation (CUT&Tag) analysis was carried out in *CEBPG*-NC A2780 cells with an anti-CEBPG antibody (12997-1-AP, Proteintech, China) by HaploX (China).

### Luciferase assay

A2780 OC cells were transfected with 100 ng PLG-*SLC7A11* plasmid, PLG-*SL*C7A11-Mut plasmid or PLG plasmid with 200 ng *CEBPG* plasmid or negative control plasmid. An analysis of firefly luciferase activity versus Renilla luciferase activity was carried out by co-transfecting the cells with 20 ng Renilla plasmid. A dual luciferase reporter system (Yeasen Biotechnology, China) was used to measure Luciferase activity 48 h after the transfection.

### Animal tumor model

For the animal experiments, the Animal Care and Use Committee of Xinhua Hospital approved them. From Slac Experimental Animal Center (Shanghai, China), six-week-old female athymic nude mice were obtained and randomly divided into different groups (n = 4). A total of 5 × 10^5^ objective OC cells (*CEBPG*-Sh1 VS *CEBPG*-NC A2780 cells or *CEBPG*-Sh1 + *SLC7A11* VS *CEBPG*-Sh1 A2780 cells) were injected into the nude mice's left ovary parenchyma. After 12 weeks, the tumors were excised after euthanasia of mice, and the total tumor burden and tumor weight were analyzed.

### Reagents

Erastin (S7242), Z-VAD-FMK (S7023), 3-methyladenine (S2767) and disulfiram (S1680) were obtained from Selleck. Ferrostatin-1 (SML0583-5MG) was obtained from Merck.

### Statistical analysis

At least three replications of each experiment were performed. Statistical comparisons were conducted between the control and experimental groups using paired t-tests or one-way analysis of variances. Statistical analyses were performed on three independent samples. A p value of 0.05 was used as the threshold for statistical significance. All data were analyzed using IBM SPSS Statistics. Based on Kaplan–Meier analysis (K-M), survival analysis was performed.

## Results

### CEBPG was associated with progression and prognosis of OC in humans

**Fig. 1:**
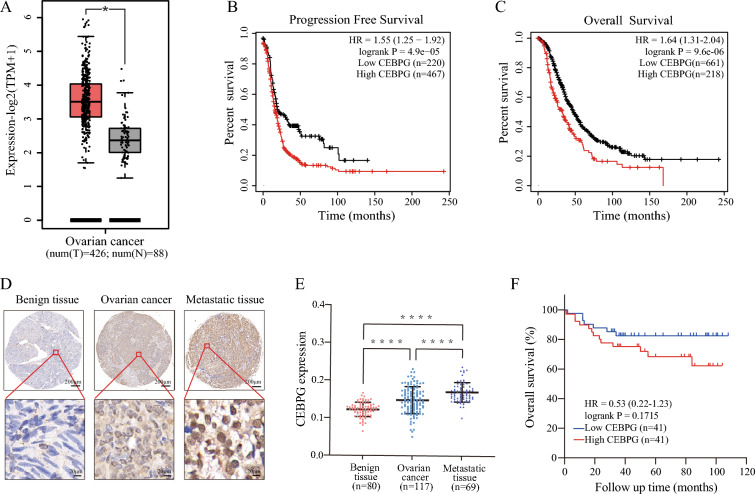
CEBPG was associated with progression and prognosis of OC in humans. **A** The expression of *CEBPG* in OC tissues (red) and normal ovarian tissues (gray) in the TCGA database. Kaplan–Meier curves of PFS **B** and OS **C** in the TCGA database. **D** Typical immunohistochemical images of TMA stained with CEBPG. Scale bars, 200 μm and 20 μm. **E** Quantification of *CEBPG* expression by IHC analysis in different tissues. **F** The OS of patients with low/high *CEBPG* expression based on IHC analysis. ****P < 0.0001

We first measured *CEBPG* expression levels in TCGA OC samples to elucidate its molecular characteristics. It was found that OC tissues expressed higher levels of *CEBPG* than benign ovarian tissues (Fig. 1A). Furthermore, based on TCGA data, high *CEBPG* expression was associated with poor progression-free survival (PFS) and overall survival (OS) (Fig. 1B, C).

Following this, benign ovarian tissues (n = 80), ovarian tumor tissues (n = 117) and peritoneal metastatic tumor tissues (n = 69) were fabricated into tissue microarrays (TMA) and analyzed for CEBPG protein expression with IHC. A higher expression level of *CEBPG* was observed in OC tissues than that in benign ovarian tissue (Fig. 1D, E). In addition, when compared with primary OC tissues, *CEBPG* expression was also elevated in peritoneal metastatic tumor tissues. In order to evaluate the significance of CEBPG in the prognosis of OC patients, we used the median of *CEBPG* expression level in IHC as the cut-off value to divide OC tissues into high-expression group and low-expression group. Consistent with the TCGA data, a trend was observed in which a high CEBPG level was apparently associated with poor OS, although there was no statistically significant difference between patient groups (Fig. 1F).

Hence, CEBPG could be regarded as a potential prognostic biomarker due to its high predictive value in OC. Additionally, CEBPG may contribute to the progression of OC based on these findings.

### Knockdown of *CEBPG* inhibited OC cell proliferation, migration and invasion

**Fig. 2:**
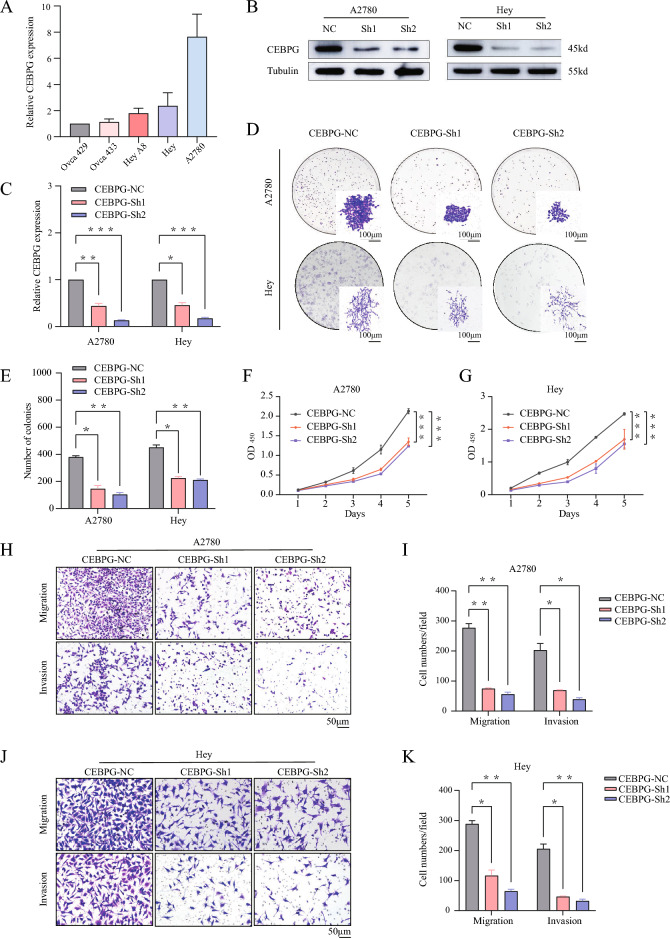
Knockdown of *CEBPG* inhibited OC cell proliferation, migration and invasion. **A** Analysis of *CEBPG* expression in different OC cell lines by qRT-PCR. **B** and **C** Western blotting and qRT‒PCR analysis of *CEBPG* expression in *CEBPG*-knockdown A2780 and Hey cells (*CEBPG*-Sh1 and *CEBPG*-Sh2) and negative control cells (control vector, *CEBPG*-NC). **D** and **E** The colony formation assays of different OC cell lines were analyzed. Scale bar: 100 μm. **F** and **G** Different OC cell lines have been tested for viability. **H–K** A comparison of migration and invasion assays between *CEBPG*-NC and *CEBPG*-knockdown OC cell lines was performed. Scale bar: 50 μm. *P < 0.05, **P < 0.01, ***P < 0.001

In order to assess what role CEBPG plays in the development of OC, a panel of OC cell lines were tested for *CEBPG* expression levels using qRT‒PCR (Fig. 2A). Following that, *CEBPG* was knocked down in A2780 and Hey cell lines with relatively high *CEBPG* levels. We confirmed the efficacy of gene knockdown in the *CEBPG* knockdown (Sh1 and Sh2) cell lines relative to the negative control (NC) cell lines via Western blotting and qRT‒PCR detection of *CEBPG* expression (Fig. 2B, C). Then, the colony formation assay results indicated that the anchorage-dependent growth of A2780 and Hey OC cells was significantly inhibited when *CEBPG* was knocked down (Fig. 2D, E). In addition, CCK8 assays revealed that growth inhibition by *CEBPG* knockdown was significantly correlated with proliferation suppression in A2780 and Hey OC cells (Fig. 2F, G). Consistent with these findings, transwell assays showed that the ability of cell migration and invasion in A2780 and Hey cells were also attenuated when *CEBPG* was knocked down (Fig. 2H–K). And the attenuation of cell migration and invasion ability was related to the increased expression of E-cadherin in OC cells (Additional file 1: Fig. S1A, B). In view of this, we speculated that CEBPG promoted the metastasis of ovarian cancer cells in part by regulating the Epithelialmesenchymal transition (EMT) pathway. Overall, knockdown of *CEBPG* inhibited various malignant behaviors involved in OC progression in cultured cells.

### CEBPG plays a crucial role in regulating ferroptosis in OC

**Fig. 3:**
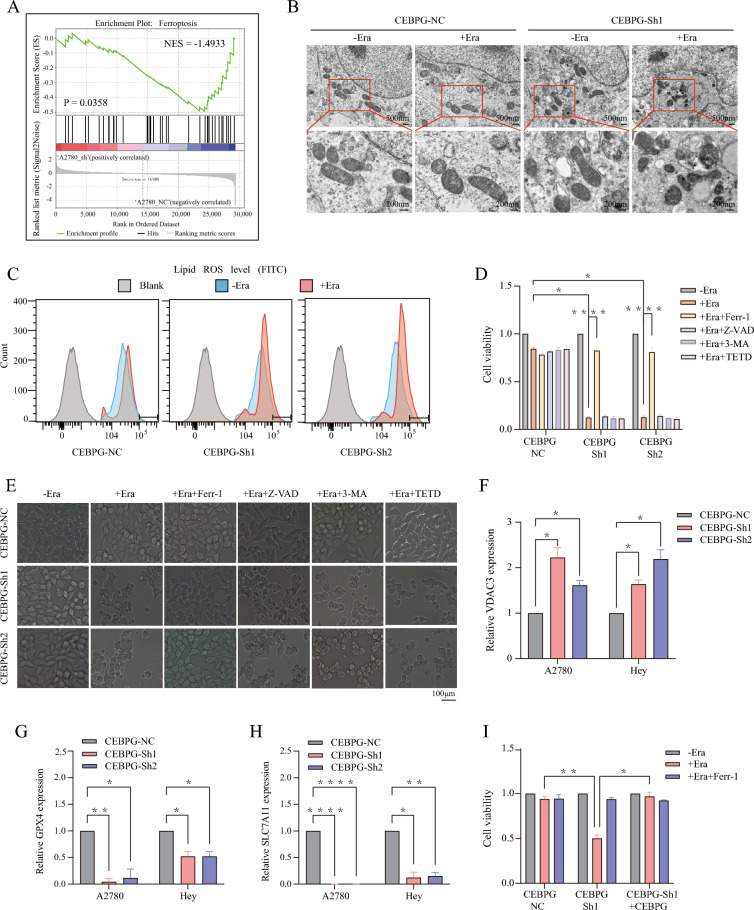
In OC, CEBPG plays a crucial role in regulating ferroptosis. **A** Gene expression analysis of A2780-*CEBPG*-Sh1 and A2780-*CEBPG*-NC cells. The signature was defined by the genes whose expression had changed significantly. **B** A TEM analysis of *CEBPG*-Sh1 A2780 cells was carried out after they had been treated with erastin (Era). Scale bars: 500 μm and 200 μm. **C** Lipid peroxidation was assessed by flow cytometry after DCFH-DA staining in *CEBPG*-knockdown and *CEBPG*-NC A2780 cells. **D** Cell viability was measured after treatment with Era alone, Era plus Ferr-1, Era plus Z-VAD, Era plus 3-MA or Era plus TETD in *CEBPG*-knockdown and *CEBPG*-NC A2780 cells. **E** Representative phase contrast images of *CEBPG*-knockdown A2780 cells treated with Era alone, Era plus Ferr-1, Era plus Z-VAD, Era plus 3-MA or Era plus TETD. Scale bar: 100 μm. **F**–**H** qRT‒PCR analysis of *VDAC3*, *GPX4* and *SLC7A11* expression in *CEBPG*-knockdown A2780 and Hey cells **I** *CEBPG*-knockdown A2780 cells with or without *CEBPG* reexpression were assessed for cell viability using Era alone or Era plus Ferr-1. *P < 0.05, **P < 0.01, ****P < 0.0001

To further elucidate the molecular mechanism of CEBPG in OC, we performed mRNA sequencing in A2780 cells with or without *CEBPG* knockdown. As a result of *CEBPG* knockdown, 162 genes were upregulated and 302 genes were downregulated (Additional file 1: Fig. S2A, B). Then a gene set enrichment analysis (GSEA) was performed, which showed that genes differentially expressed in the two groups were enriched in ferroptosis genes (Fig. 3A and Additional file 1: Fig. S2C).

Moreover, the result of transmission electron microscopy (TEM) revealed that *CEBPG*-knockdown A2780 OC cells treated with the ferroptosis inducer erastin (Era) displayed shrunken mitochondria and an increased membrane density, a hallmark of ferroptosis [18], compared with that in cells without Era treatment, but *CEBPG*-NC A2780 OC cells treated with Era did not exhibit the same phenotype (Fig. 3B and Additional file 1: Fig. S1D). This suggested that knockdown of *CEBPG* promoted ferroptosis in OC cells. Given that the excessive accumulation of intracellular ROS is one of the characteristics of cell ferroptosis. Here, different groups of A2780 cells were stained with DCFH-DA fluorescent probes, and the amount of intracellular ROS was finally measured by detecting the fluorescence intensity of the stained cells by flow cytometry. Flow cytometry results showed that knockdown of *CEBPG* significantly increased Era-induced lipid peroxidation in A2780 OC cells (Fig. 3C). Functionally, CCK8 assays and microscopy also showed that knockdown of *CEBPG* significantly promoted Era-triggered cell death in OC cells, and this effect was prevented by treatment with Ferr-1, a ferroptosis inhibitor. Conversely, inhibitors of other types of cell death, including apoptosis (Z-VAD-FMK), autophagy (3-methylademine, 3-MA) and pyroptosis (disulfiram, TETD), all failed to inhibit the Era-induced increase in cell death in *CEBPG* knockdown OC cell lines (A2780 and Hey; Fig. 3D, E, Additional file 1: Fig. S2E, F), indicating that Era-triggered cell death in *CEBPG*-knockdown OC cells was not involved in apoptosis, autophagy or pyroptosis. Moreover, the results of qRT-PCR showed that the key ferroptosis positive regulator genes *VDAC3* was up-regulated and the negative ferroptosis regulator gene *GPX4* and *SLC7A11* were down-regulated after *CEBPG* knockdown in A2780 and Hey cell lines (Fig. 3F–H) [19].

Furthermore, A2780 cells with stable *CEBPG* knockdown were subsequently re-expressed with *CEBPG*, resulting in the significant reversion of Era-induced ferroptosis (Fig. 3I). Collectively, these evidences suggested that knockdown of *CEBPG* stimulated ferroptosis in OC cells.

### Knockdown of *CEBPG* inhibited OC progression partially by promoting the transcription of *SLC7A11*

**Fig. 4:**
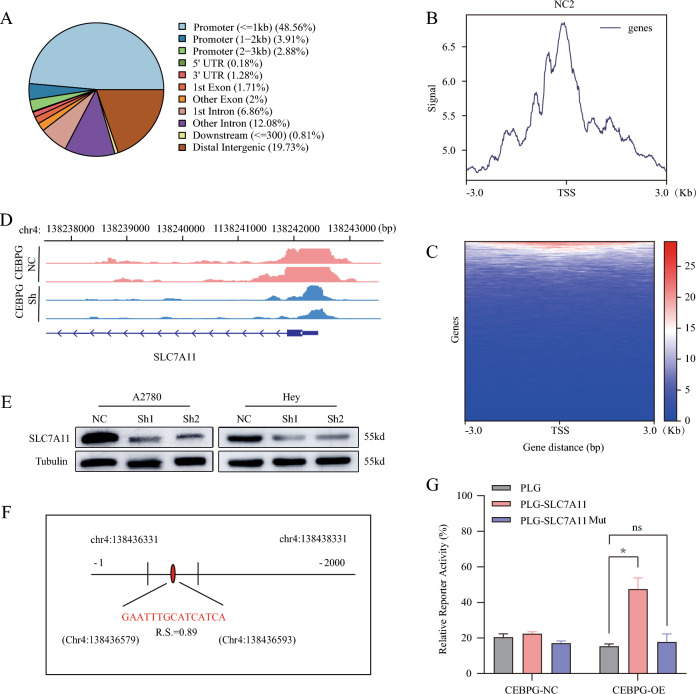
Knockdown of *CEBPG* inhibited the transcription of *SLC7A11* in OC cells. **A** The genomic distribution of CUT&Tag peaks in A2780 cells. **B** and **C** The distribution of mapped *SLC7A11* peak locations within 3 kb upstream and downstream of TSSs in genes with *SLC7A11*-bound promoters. **D** The results of CUT&Tag-seq showed the binding site domain for CEBPG in the *SLC7A11* gene promoter region in A2780 cells. **E** SLC7A11 protein expression levels in different cell lines were determined by immunoblotting.** F** The JASPAR website was used to scan the CEBPG-bound promoter and predict one potential binding site (GAATTTGCATCATCA) for *CEBPG*. The relative score (RS) = 0.890 for the motifs. **G** Dual luciferase reporter assay with the promoter of *SLC7A11* (PLG-*SLC7A11*), the mutated promoter of *SLC7A11* (PLG-*SLC7A11* Mut) or the control promoter of *SLC7A11* (PLG) in *CEBPG*-overexpressing (*CEBPG*-OE) A2780 cells. *P < 0.05, ns: no significance

An analysis of high-throughput CUT&Tag data was performed to gain a better understanding of *CEBPG*-regulated ferroptosis in OC. The identification of 13,430 differential peaks associated with genome-binding was carried out, and 55.35 percent of these differential peaks were found in gene promoter regions—from – 3– + 3 kb with respect to the transcriptional start site (TSS)—and exhibited a high density around the TSS (Fig. 4A–C). Notably, visual analysis with IGV showed that there was a CEBPG binding peak in the promoter of *SLC7A11* (peak 10072), a key suppressor of ferroptosis (Fig. 4D). In addition, previous mRNA sequencing results showed that *SLC7A11* expression was significantly down-regulated in *CEBPG*-knockdown A2780 cells (Additional file 1: Fig. S2C), which was further confirmed that by Western blotting in *CEBPG*-knockdown A2780 and Hey OC cells (Fig. 4E). Taken together, our data indicated that CEBPG might regulate OC cell ferroptosis through transcriptional control of *SLC7A11*.

To test above hypothesis, we searched JASPAR (http://jaspar.genereg.net) to find putative binding sites in the *SLC7A11* promoter. JASPAR analysis reveals potential binding sites which are quantitatively scored based on the probability that each nucleotide is observed at each position in the binding motif versus the consensus sequence for known binding sites. Sequence analysis by JASPAR showed 6 putative binding sites totally (Additional file 1: Fig. S3A). As for the sequence with the highest relative score (R.S.), its location was in the promoter upstream of the transcription start site, and its R.S. were GAATTTGCATCATCA, − 262–− 248 bp, and 0.89, respectively (Fig. 4F). Based on this potential binding site, a luciferase reporter assay was conducted in A2780 cells co-transfected with *CEBPG* plasmids containing the full‐length promoter of *SLC7A11*, *CEBPG* plasmids containing the binding region mutant of the *SLC7A11* promoter or pGL as a control vector, respectively. The results showed extremely different levels of *SLC7A11* promoter activity in these cell groups (Fig. 4G). Therefore, these findings indicated that CEBPG upregulated the expression of *SLC7A11* by enhancing its transcription.

**Fig. 5:**
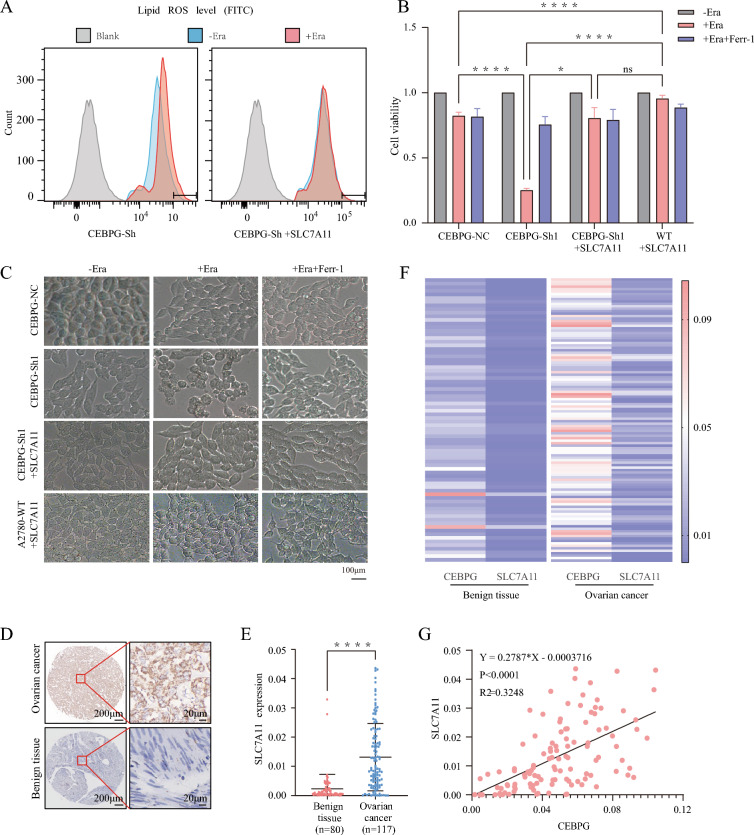
The knockdown of *CEBPG* inhibited OC progression partially by reducing *SLC7A11* expression and activating ferroptosis. **A** The lipid peroxidation of *CEBPG*-knockdown A2780 cells with or without reexpression of *SLC7A11* was assessed by flow cytometry after DCFH-DA staining. **B** The viability of *CEBPG*-knockdown A2780 cells with or without *SLC7A11* reexpression was assessed after treatment with Era alone or with Era plus Ferr-1. **C** Representative phase contrast images of *CEBPG*-knockdown A2780 cells treated with Era alone or combined with Ferr-1 when *SLC7A11* was reexpressed. Scale bar: 100 μm. **D** Representative images of SLC7A11 staining in benign ovarian cyst and OC tissues. Scale bars, 200 μm and 20 μm. **E** Quantification of *SLC7A11* expression in different tissues by IHC analysis. **F** Heatmap of *CEBPG* and *SLC7A11* expression as determined by IHC analysis in different tissues. **G** The correlation of SLC7A11 expression levels with CEBPG levels was analyzed in a TAM. *P < 0.05, ****P < 0.0001, ns: no significance

To further validate the function of CEBPG in regulating SLC7A11-involved ferroptosis. More rescue experiments were conducted. Firstly, Era-induced lipid peroxidation was inhibited by re-expression of *SLC7A11* in *CEBPG*-Sh1 A2780 OC cells (Fig. 5A). In addition, restoration of SLC7A11 almost completely suppressed Era-triggered cell death in *CEBPG*-knockdown A2780 and Hey OC cells (Fig. 5B, C; Additional file 1: Fig. S3B). Correspondingly, OC tissues had a higher level of SLC7A11 than benign ovarian tissues in the TMA, as evaluated by IHC staining (Fig. 5D–F). Importantly, a positive correlation was found between *CEBPG* and *SLC7A11* expression levels in TMA (R^2^ = 0.3248, P < 0.0001; Fig. 5G).

**Fig.6:**
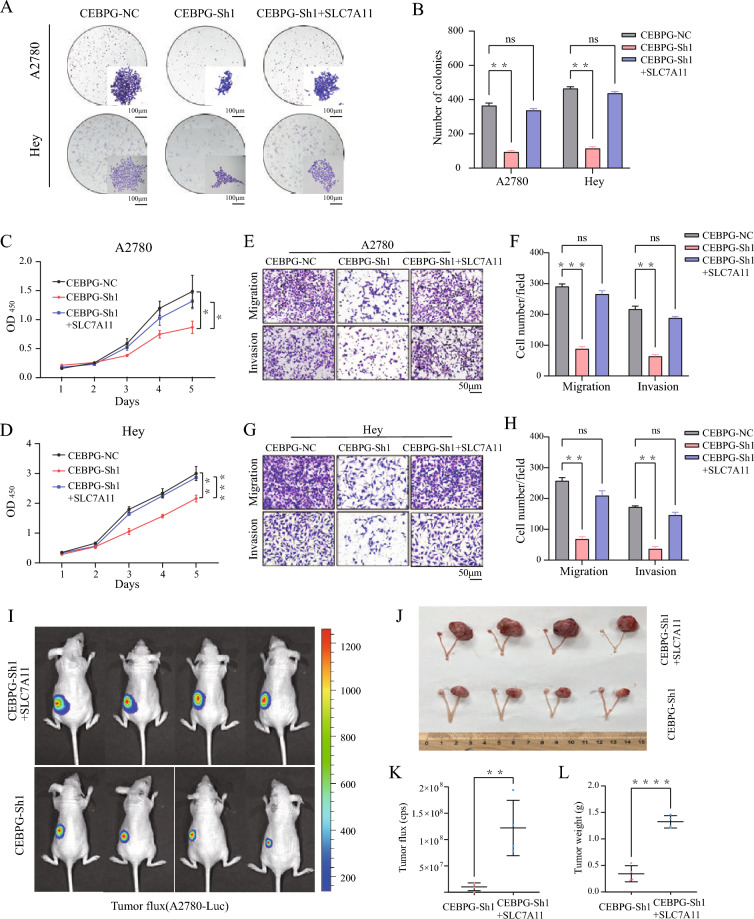
Decreased CEBPG inhibited OC progression at least partly through suppression of *SLC7A11* both in vitro and in vivo. Colony formation **A** and **B** and growth **C** and **D** of CEBPG-decreased A2780 and Hey cells, with or without *SLC7A11* reexpression, were measured. Scale bar: 100 μm. **E**–**H** The migration and invasion assays of OC cell lines, with or without *SLC7A11* reexpression, were analyzed, Scale bar: 50 μm. **I** *CEBPG*-knockdown A2780 cells, with or without SLC7A11 reexpression, were subcutaneously injected into the ovaries of nude mice. Images of orthotopic ovarian tumors in mice. **J** Pictures of the removed tumors. **K** Luciferase expression was detected in model mice. **L** Analysis of tumor weight in the two groups. *P < 0.05, **P < 0.01, ***P < 0.001, ****P < 0.0001, ns: no significance

Moreover, since re-expression of *SLC7A11* could reverse Era-triggered ferroptosis in *CEBPG* knockdown OC cells, we next explored whether and to what extent SLC7A11 could restore the malignant behavioral capacity of *CEBPG* knockdown OC cells. We stably transfected *CEBPG*-knockdown A2780 and Hey OC cells with a lentivirus expressing *SLC7A11* or a control vector. Re-expression of *SLC7A1*1 in stably *CEBPG*-knockdown OC cells partially restored the ability of cell growth and proliferation inhibited by *CEBPG* knockdown, as judged by colony formation assays and CCK8 assays (Fig. 6A–D). In addition, the transwell assays revealed that the migrative and invasive ability of *CEBPG*-decreased OC cells was significantly promoted by *SLC7A11* re-expression (Fig. 6E–H). In vivo, *CEBPG* knockdown–induced inhibition of xenograft tumor growth was also significantly rescued by *SLC7A11* restoration (Fig. 6I–L), consistent with that in vitro. Together, our results indicated that the function of *CEBPG* suppression in OC progression inhibition was caused partially through repression of *SLC7A11.*

In summary, our data showed that in the absence of CEBPG, ferroptosis was primarily promoted by down-regulating the expression of *SLC7A11.* Further, the inhibition of OC progression due to decrease of CEBPG was also associated with SLC7A11 because that the malignant behaviors of OC cells could partially be restored by *SLC7A11* re-expression and increased levels of ferroptosis.

### CEBPG promoted the progression of OC in vivo.

**Fig. 7:**
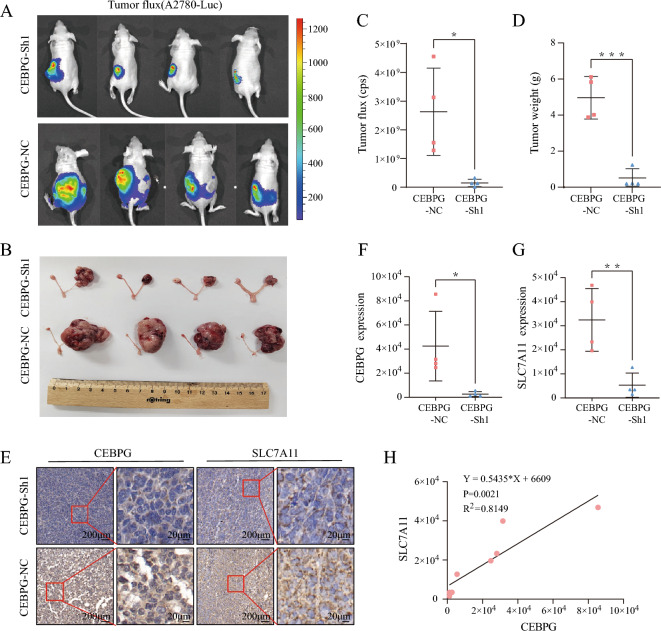
CEBPG promoted OC progression in vivo. **A** and **B** Images of orthotopic ovarian tumors in mice. **C** Luciferase expression was detected in model mice. **D** Analysis of tumor weight in the two groups. **E** Representative images of immunohistochemical staining of CEBPG and SLC7A11 in orthotopic ovarian tumor tissues. Scale bars: 200 μm and 20 μm. **F** and **G** CEBPG and SLC7A11 levels in orthotopic ovarian tumor tissues were analyzed. **H** The correlation of SLC7A11 expression levels with CEBPG levels was analyzed in orthotopic ovarian tumor tissues. *P < 0.05, **P < 0.01, ***P < 0.001, ns: no significance

For the purpose of validating CEBPG function in vivo, we developed a mouse orthotopic model of OC using *CEBPG*-Sh1 and *CEBPG*-NC A2780 OC cells. Longitudinal bioluminescence imaging was performed to assess the tumor burden, and orthotopic tumors were excised for final analysis. The results showed that *CEBPG*-Sh1 A2780 cells grew much slower and developed smaller tumors than control cells, similar to what we observed in vitro (Fig. 7A–D).

Then, we analyzed the levels of CEBPG and SLC7A11 in these orthotopic tumor tissues by IHC staining. Remarkably, tissues of all tumors derived from *CEBPG*-NC A2780 cells had significantly higher levels of *CEBPG* and *SLC7A11* expression than those derived from *CEBPG*-Sh1 A2780 cells (Fig. 7E–G), and *SLC7A11* expression level correlated positively with *CEBPG* expression level in orthotopic ovarian tumor tissues (R^2^ = 0.8149, P < 0.0001; Fig. 7H). Moreover, we extracted protein and RNA from these tumor tissues to examine the expression levels, the results of Western Blotting and qRT-PCR showed that knockdown of *CEBPG* in mice also reduced the expression of *SLC7A11* (Additional file 1: Fig.S4A–C). Taken together, our data further indicated that CEBPG promoted the progression of OC in vivo.

## Discussion

In recent years, CEBPG hyperactivation has been reported in numerous studies as a factor contributing to the progression of cancer; however, the exact mechanism of CEBPG remains a mystery in OC. In addition, accumulating evidence suggests that SLC7A11 promotes cancer progression in part by inhibiting ferroptosis, a form of programmed cell death caused by excessive lipid peroxidation. As well, it has been shown that SLC7A11 may contribute to cancer progression in part by inhibiting ferroptosis, an excessive lipid peroxidation-induced form of programmed cell death. In this study, we investigated *CEBPG* and *SLC7A11* expression in OC patients and evaluated their correlation with long-term survival. In a clinical analysis, *CEBPG* expression correlated significantly with *SLC7A11* expression, indicating poor prognosis worsening. CEBPG-associated promotion of OC appeared to be mainly mediated by SLC7A11-mediated ferroptosis inhibition. For the purpose of testing this concept, additional in vitro validation studies were conducted to further assess CEBPG-SLC7A11-ferroptosis axis' potential as OC biomarker targets.

A leucine zipper transcription factor known as CEBPG is vital to a number of biological processes [20]. Based on analysis of CEBPG in mouse fibroblasts, it was shown that CEBPG promotes cell proliferation while suppressing senescence [21]. Additionally, *CEBPG* acts as a regulator gene for the progression of many types of cancer, such as acute myeloid leukemia, lung cancer, and esophageal cancer [7–9]. It is unknown, however, how CEBPG regulates and functions biologically. In agreement with these observations, we found CEBPG could enhance ovarian tumor cell proliferation, invasion, and migration. As shown in the orthotopic ovarian tumorigenesis assay, knockdown of *CEBPG* significantly reduced tumor growth in nude mice. Then, we have confirmed that CEBPG acted as an important regulator of ferroptosis in OC progression. Moreover, in patients with OC, we found that *CEBPG* overexpression was associated with poor prognosis.

SLC7A11 regulates cellular lipid peroxidation and suppresses ferroptosis. SLC7A11 has been shown to promote cancer progression by inhibiting ferroptosis in recent studies [22–24]. For example, P53 mutant 3KR inhibits *SLC7A11* expression, causing ferroptosis, while retaining partial tumor suppressor functions [22]. When SLC7A11 is inhibited, BAP1 triggers ferroptosis to partially inhibit cancer progression [23]. Moreover, posttranslational regulation of *SLC7A11* by RBMS1 mediates ferroptosis in lung cancer [24]. Here, we found that the activation of CEBPG could also regulate transcription of *SLC7A11* by binding to its promoter region, which is related to ferroptosis inhibition. IHC analyses showed that SLC7A11 expression levels correlated significantly with CEBPG levels, indicating that *SLC7A11* and *CEBPG* expression levels were positively correlated. The expression of *CEBPG* in OC cell lines also substantially increased *SLC7A11* expression. Interestingly, based on our dual-luciferase reporter assays analyses, we showed that CEBPG directly binds to the promoter region of *SLC7A11* and controls its expression. In OC, these results confirmed CEBPG and SLC7A11 interaction and revealed their underlying role in prognosis.

Ferroptosis is an iron-dependent form of cell death that kills cells by oxidatively disrupting the intracellular plasma membrane [25]. In general, both traditional and targeted cytotoxic drugs work by inducing cancer cell death in order to slow or stop tumor growth, while in most cases, anticancer drugs kill tumor cells by triggering apoptosis [26]. Antineoplastic drugs and compounds are increasingly reported to trigger ferroptosis, that is, chemotherapy drugs kill tumor cells through ferroptosis in addition to apoptosis [27]. As reported, cisplatin, one of the first-line chemotherapy drugs for OC patients, could also induce ferroptosis in tumor cells [28]. Further, it was recently reported that *KRAS*, which was found mutation/amplification in nearly 13.9% of ovarian cancers [29], may play a role in ferroptosis and lipid biosynthesis [30], while other studies found that mutant *KRAS* activates the NRF2 antioxidant pathway, in which NRF2 activated glutaminolysis and glutamine deprivation resulted in reduced GPX4 levels [31]. It is an interesting aspect that NRF2 plays a significant role in lipid peroxidation and ferroptosis [32]. In OC, the role of glutamine metabolism in regulating GPX4 might be one of the future areas of research in the field. Taken together with our data, *CEBPG*-knockdown cells showed increased sensitivity to ferroptosis inducers, also pointing towards potential usage for CEBPG in OC treatment.

As mentioned above, CEBPG is a CEBP family member. In contrast to classical CEBP family members which typically contain DNA-binding domains at C-termini, activation domains at N-termini, and regulatory domains, CEBPG does not possess a transactivation domain [33, 34]. Therefore, rather than forming stable homodimers, CEBPG preferentially heterodimerizes with other molecules to function [8, 21]. In the present study, we showed that *CEBPG* overexpression led to a significant increase in *SLC7A11* expression, and that CEBPG can regulate its transcription by binding to the promoter region. Nevertheless, further investigation is needed to determine whether CEBPG has this effect alone or in conjunction with other molecules. It is worth mentioning that CEBPG can enhance PI3K-AKT signaling, but it is not clear whether CEBPG also regulates ferroptosis through this pathway. Therefore, we would like to utilize PI3K/AKT inhibitors in this current experimental set up and see how that works to regulate the role of CEBPG in ferroptosis in the future.

## Conclusions

**Fig. 8:**
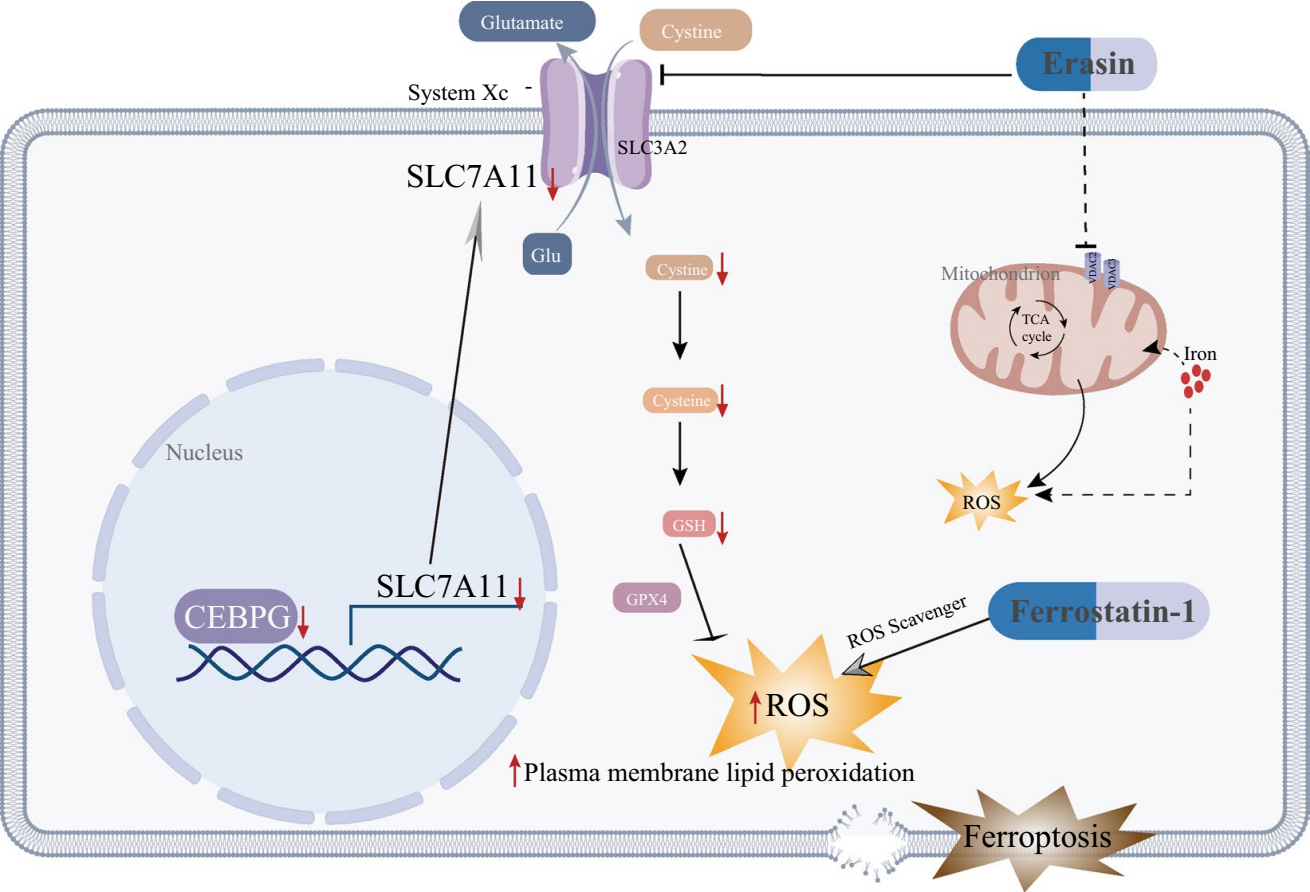
Schematic diagram of how CEBPG regulates OC cell ferroptosis through transcriptional control of SLC7A11

In summary, we found that CEBPG promoted ovarian tumor cell proliferation and inhibited ferroptosis in vitro and accelerated tumor growth in vivo, and the promotion of ovarian cancer by CEBPG is partially dependent on SLC7A11 (Fig. 8). We reported for the first time that CEBPG enhanced the transcription of *SLC7A11* to mediate ferroptosis in OC. At the clinical level, we observed that CEBPG expression was also associated with poor outcomes of OC patients. Thus, our study proved CEBPG as an important transcriptional regulator of ferroptosis as well as a prognostic factor with potential therapeutic values.

## Supplementary Information

**Additional file 1: Figure S1.** Knockdown of CEBPG inhibited OC migration. **Figure S2.** CEBPG plays a crucial role in regulating ferroptosis in OC. **Figure S3.** The knockdown of CEBPG inhibited OC progression partially by reducing *SLC7A11* expression and activating ferroptosis. **Figure S4.** CEBPG promoted the progression of OC in vivo. **Table S1.** The shRNA sequences targeting *CEBPG*. **Table S2.** Primer sequence.

## Data Availability

Upon reasonable request, the corresponding author will provide access to any data used or analyzed during this study.

## Abbreviations

OC: Ovarian cancer
CEBPG: CCAAT/enhancer binding protein gamma
SLC7A11: Solute carrier family 7 member 11
TCGA: The cancer genome atlas
IHC: Immunohistochemical staining
GSH: Glutathione
GPX4: Glutathione peroxidase 4
OS: Overall survival rate
TMA: Tissue microarray
qRT-PCR: Quantitative real-time PCR
CCK8: Cell counting kit 8
EMT: Epithelialmesenchymal transition
TEM: Transmission electron microscopy
Era: Erastin
3-MA: 3-methyladenine
TETD: Disulfiram
Ferr-1: Ferrostatin-1
PFS: Progression-free survival
GSEA: Gene set enrichment analysis
TSS: Transcriptional start site
