# Supplementary material for: CEBPG suppresses ferroptosis through transcriptional control of SLC7A11 in ovarian cancer
Source: J Transl Med. 2023 May 20;21:334. doi: 10.1186/s12967-023-04136-0 (PMC10199564; doi:10.1186/s12967-023-04136-0)
Supplement: Supplementary file 1 — Additional file 1: Figure S1. Knockdown of CEBPG inhibited OC migration. Figure S2. CEBPG plays a crucial role in regulating ferroptosis in OC. Figure S3. The knockdown of CEBPG inhibited OC progression partially by reducing SLC7A11 expression and activating ferroptosis. Figure S4. CEBPG promoted the progression of OC in vivo. Table S1. The shRNA sequences targeting CEBPG. Table S2. Primer sequence. [file 12967_2023_4136_MOESM1_ESM.docx]

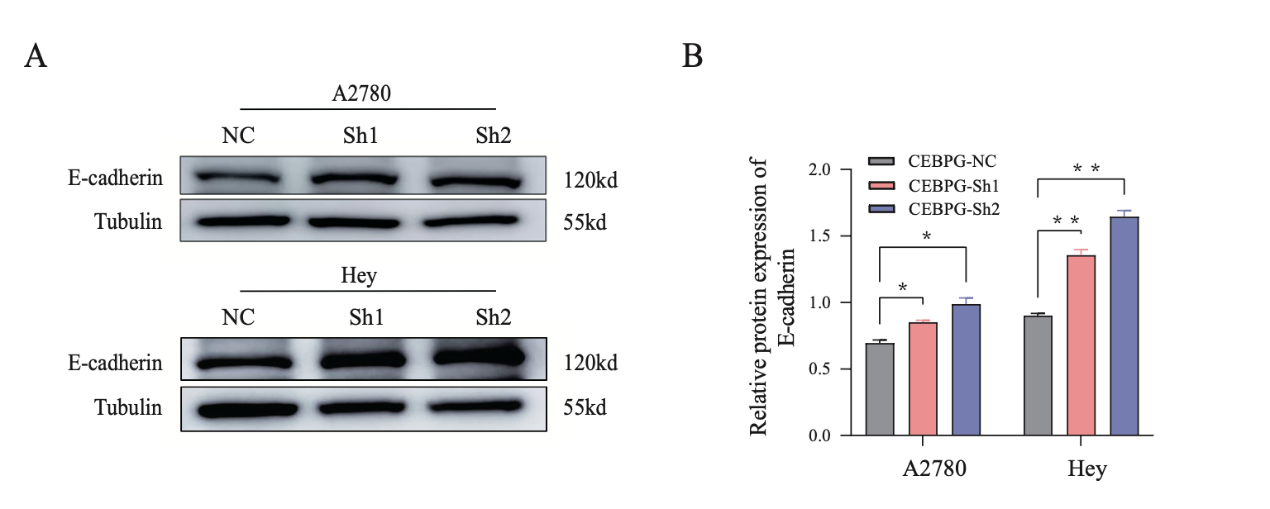


**Fig.S1** **Knockdown of *CEBPG* inhibited OC migration.**

**A** and **B** The protein expression levels of E-cadherin analyzed by Western blotting in *CEBPG*-knockdown and control A2780 and Hey OC cells. *P < 0.05, **P < 0.01.


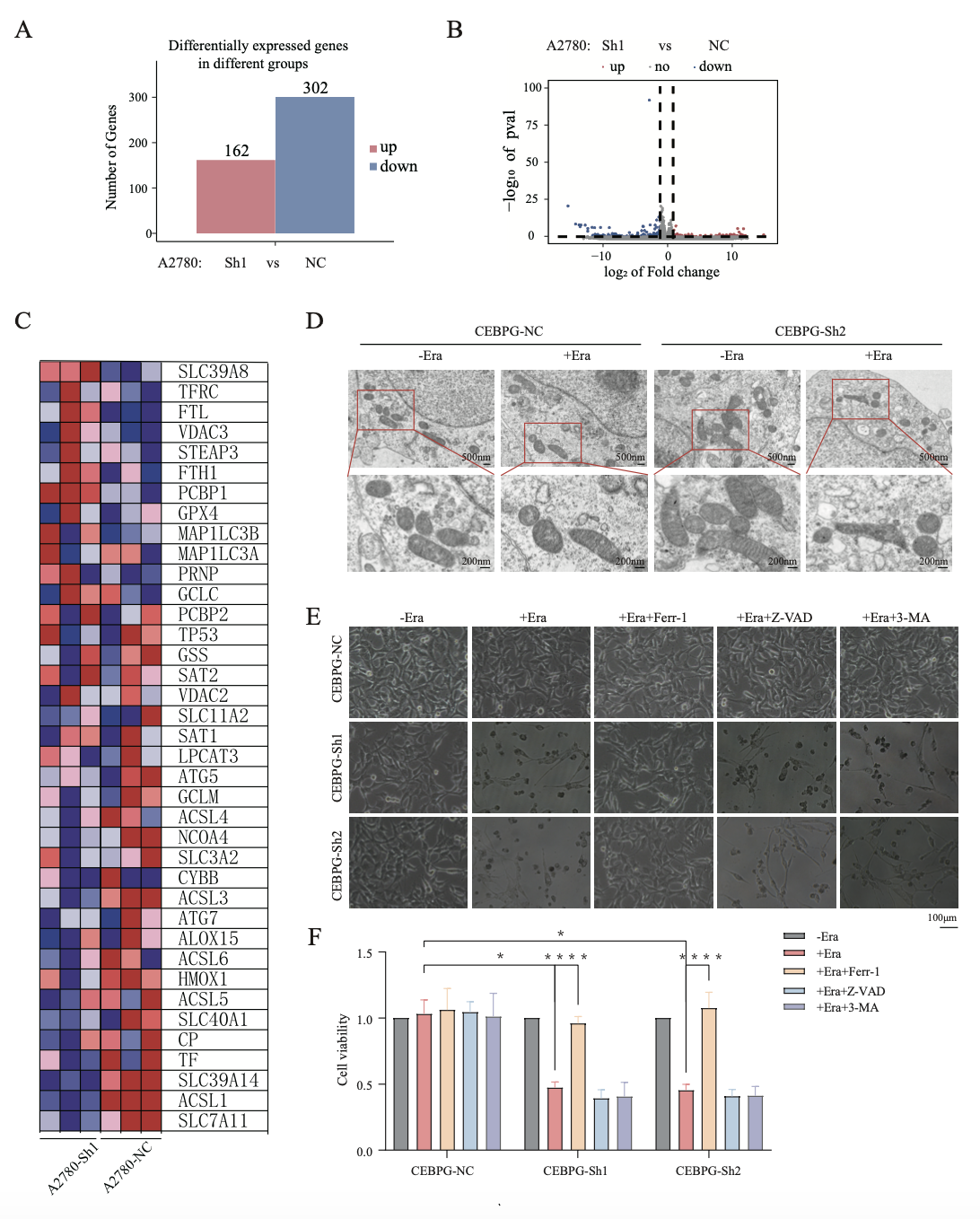


**Fig. S2 CEBPG plays a crucial role in regulating ferroptosis in OC.**

**A and B** The differential expressed genes between A2780-*CEBPG*-Sh1 and A2780-*CEBPG*-NC cells analyzed by RNA-seq. **C** The heatmap of differential expressed genes related to ferroptosis between A2780-*CEBPG*-Sh1 and A2780-*CEBPG*-NC cells. **D** TEM analysis of *CEBPG*-Sh2 and control A2780 cells with or without Era treatment, related to Fig.3B. Scale bars: 500nm and 200nm. **E** Representative phase contrast images of *CEBPG*-knockdown and control Hey cells treated with Era alone, Era plus Ferr-1, Era plus Z-VAD or Era plus 3-MA. Scale bar: 100μm. **F** Cell viability was measured after treatment with Era alone, Era plus Ferr-1, Era plus Z-VAD or Era plus 3-MA in *CEBPG*-knockdown and *CEBPG*-NC Hey cells. *P < 0.05, ****P < 0.0001.


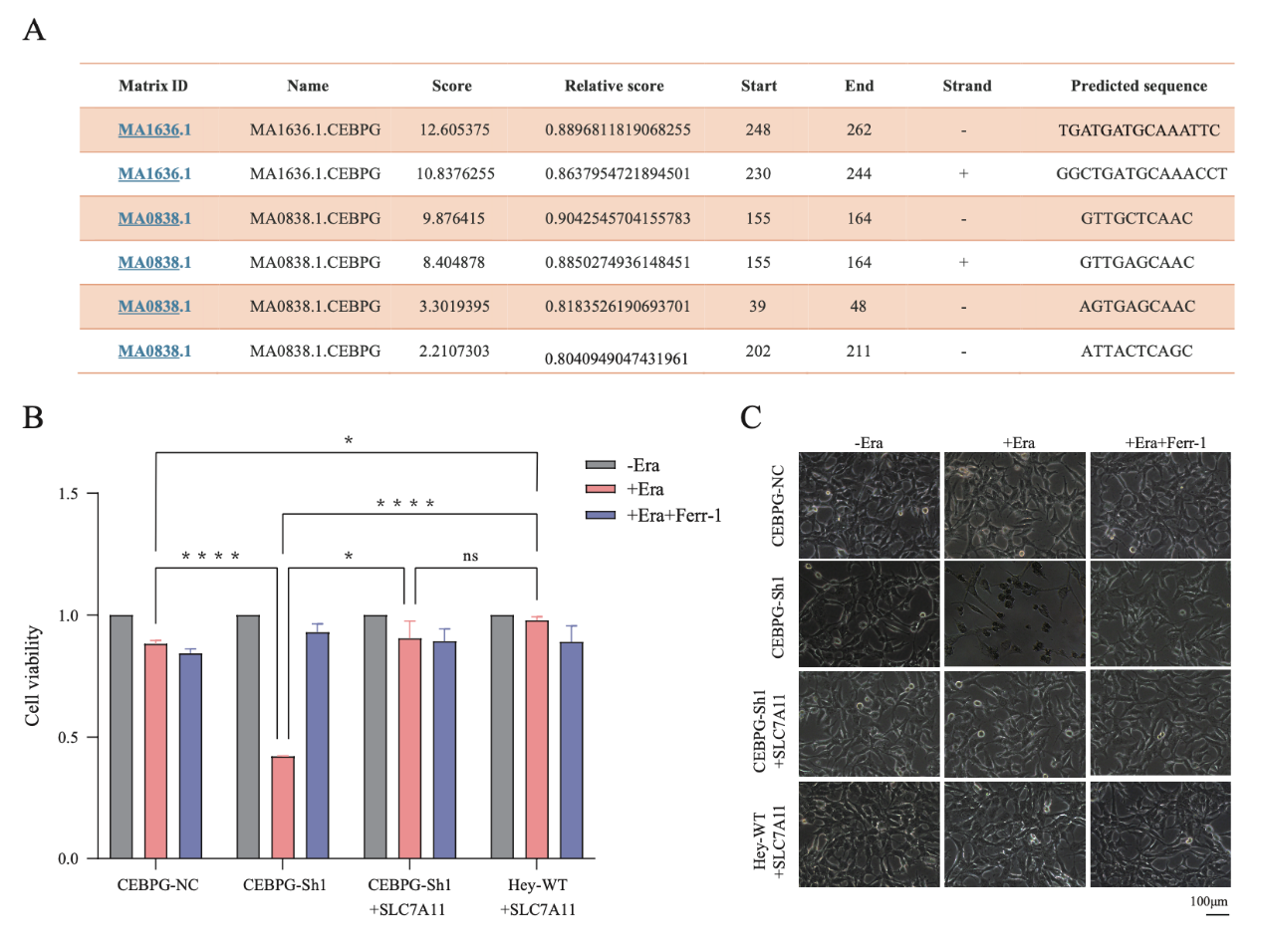


**Fig. S3 The knockdown of *CEBPG* inhibited OC progression partially by reducing *SLC7A11* expression and activating ferroptosis.**

**A** The top 6 putative binding sites in the *SLC7A11* promoter predicted in JASPAR. **B** The viability of *CEBPG*-knockdown Hey cells with or without *SLC7A11* reexpression was assessed after treatment with Era alone or with Era and Ferr-1. **C** Representative phase contrast images of *CEBPG*-knockdown Hey cells treated with Era alone or combined with Ferr-1 when *SLC7A11* was reexpressed. Scale bar: 100μm. *P < 0.05, ****P < 0.0001, ns: no significance.


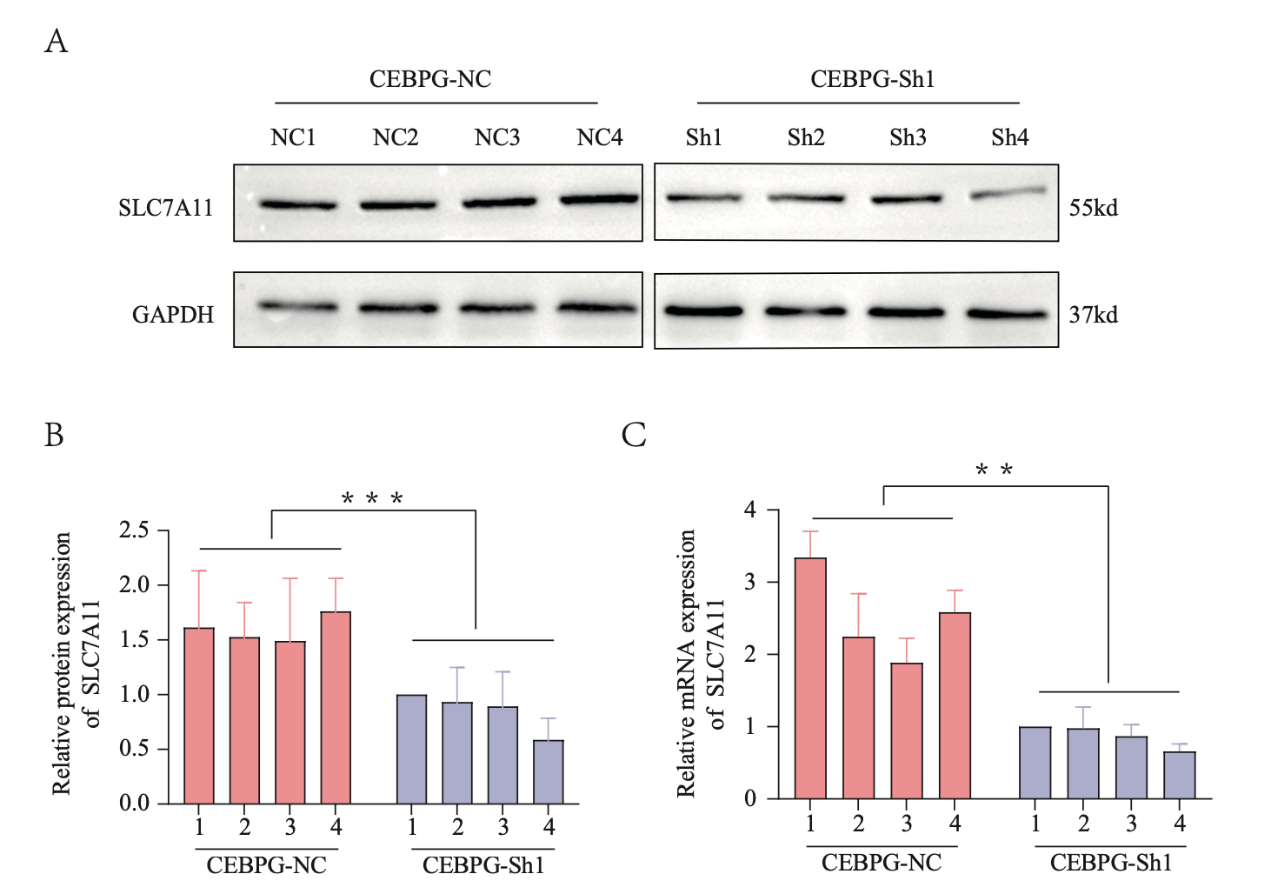


**Fig. S4 CEBPG promoted the progression of OC in vivo.**

**A** and **B** The protein expression levels of SLC7A11 analyzed by Western blotting in tumor tissues of mice seeded with *CEBPG*-knockdown or control A2780 cell. **C** The relative mRNA expression levels of *SLC7A11* analyzed by qRT-PCR in tumor tissues of mice seeded with *CEBPG*-knockdown or control A2780 cells. **P < 0.01, ***P < 0.001.


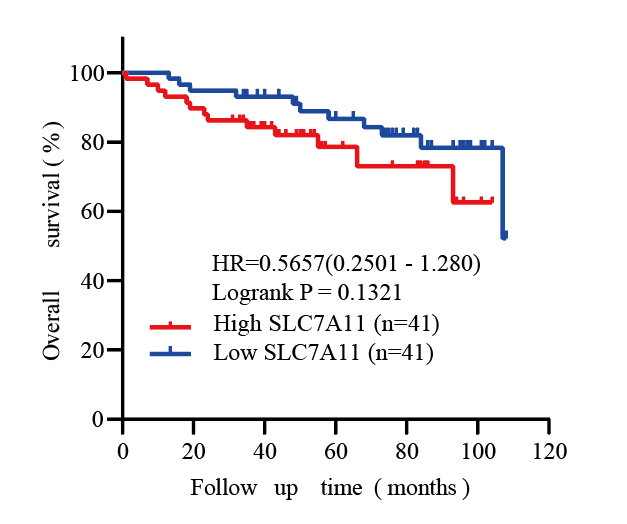


**Fig. R1 The OS of patients with low/high SLC7A11 expression based on IHC analysis.**

**Supplementary Table 1.**

**The shRNA sequences targeting *CEBPG***

| **NO.** | **Primer sequence** |
| --- | --- |
| **ShA** | **GCCCATGGATCGAAACAGT** |
| **ShB** | **GACAGCAGATGGCGACAAT** |
| **ShC** | **GAGTCAATCAGCTCAAAGA** |

**Supplementary Table 2.**

**Primer sequence**

| **Gene** | **Primer sequence** | **Application** |
| --- | --- | --- |
| ***CEBPG*** | **Forward Primer: ACTCCAGGGGTGAACGGAAT** | **qRT-PCR** |
|  | **Reverse Primer: CATGGGCGAACTCTTTTTGCT** |  |
| ***SLC7A11*** | **Forward Primer: TCTCCAAAGGAGGTTACCTGC** | **qRT-PCR** |
|  | **Reverse Primer: AGACTCCCCTCAGTAAAGTGAC** |  |
| ***GAPDH*** | **Forward Primer: GGAGCGAGATCCCTCCAAAAT** | **qRT-PCR** |
|  | **Reverse Primer: GGCTGTTGTCATACTTCTCATGG** |  |
| ***GPX4*** | **Forward Primer: GAGGCAAGACCGAAGTAAACTAC** | **qRT-PCR** |
|  | **Reverse Primer: CCGAACTGGTTACACGGGAA** |  |
| ***VDAC3*** | **Forward Primer：TTGTACCGAACACAGGAAAGAAG** | **qRT-PCR** |
|  | **Reverse Primer：CCCAGCCATAGATGGTTGGTC** |  |
